# Correction: Neutrophil DREAM come true: The not-so-impossible quest for mechanisms of neutrophil function and heterogeneity

**DOI:** 10.1084/jem.2021214112202021c

**Published:** 2021-12-28

**Authors:** John C. Gomez, Claire M. Doerschuk

Vol. 219, No. 1 | 10.1084/jem.20212141 | December 15, 2021

The authors regret that there was an error in the fifth paragraph of their Insight. Li et al. did not study platelet–neutrophil interactions in the lung microvessels, but rather in the cremaster venules. The corrected sentence appears below.

“Importantly, when mice with sickle cell disease and deficiency of DREAM were challenged with TNF-α, platelet–neutrophil interactions in cremaster venules were inhibited, blood flow was higher, and there was a small improvement in survival compared with mice with DREAM, suggesting that DREAM was required for full manifestation of vaso-occlusive events.”

The error appears in PDFs downloaded before December 21, 2021.

